# Patient and public attitudes to and awareness of clinical practice guidelines: a systematic review with thematic and narrative syntheses

**DOI:** 10.1186/1472-6963-14-321

**Published:** 2014-07-27

**Authors:** Kirsty Loudon, Nancy Santesso, Margaret Callaghan, Judith Thornton, Jenny Harbour, Karen Graham, Robin Harbour, Ilkka Kunnamo, Helena Liira, Emma McFarlane, Karen Ritchie, Shaun Treweek

**Affiliations:** 1 Division of Population Health Sciences, University of Dundee, Kirsty Semple Way, Dundee DD2 4BF, UK; 2 McMaster University, Hamilton, Canada; 3 Scottish Intercollegiate Guidelines Network, NHS Healthcare Improvement Scotland, Glasgow, Scotland; 4 National Institute for Health and Care Excellence, Manchester, UK; 5 Duodecim Medical Publications Ltd, Helsinki, Finland; 6 Health Services Research Unit, University of Aberdeen, Aberdeen, UK

**Keywords:** Clinical practice guidelines, Patient, Public, Attitudes, Awareness

## Abstract

**Background:**

Clinical practice guidelines are typically written for healthcare providers but there is increasing interest in producing versions for the public, patients and carers. The main objective of this review is to identify and synthesise evidence of the public’s attitudes towards clinical practice guidelines and evidence-based recommendations written for providers or the public, together with their awareness of guidelines.

**Methods:**

We included quantitative and qualitative studies of any design reporting on public, patient (and their carers) attitudes and awareness of guidelines written for providers or patients/public. We searched electronic databases including MEDLINE, PSYCHINFO, ERIC, ASSIA and the Cochrane Library from 2000 to 2012. We also searched relevant websites, reviewed citations and contacted experts in the field. At least two authors independently screened, abstracted data and assessed the quality of studies. We conducted a thematic analysis of first and second order themes and performed a separate narrative synthesis of patient and public awareness of guidelines.

**Results:**

We reviewed 5415 records and included 26 studies (10 qualitative studies, 13 cross sectional and 3 randomised controlled trials) involving 24 887 individuals. Studies were mostly good to fair quality. The thematic analysis resulted in four overarching themes: Applicability of guidelines; Purpose of guidelines for patient; Purpose of guidelines for health care system and physician; and Properties of guidelines. Overall, participants had mixed attitudes towards guidelines; some participants found them empowering but many saw them as a way of rationing care. Patients were also concerned that the information may not apply to their own health care situations. Awareness of guidelines ranged from 0-79%, with greater awareness in participants surveyed on national guideline websites.

**Conclusion:**

There are many factors, not only formatting, that may affect the uptake and use of guideline-derived material by the public. Producers need to make clear how the information is relevant to the reader and how it can be used to make healthcare improvements although there were problems with data quality. Awareness of guidelines is generally low and guideline producers cannot assume that the public has a more positive perception of their material than of alternative sources of health information.

## Background

Clinical practice guidelines are systematically developed tools that present recommendations and research evidence to direct appropriate healthcare throughout the world. They are typically produced for health care providers but there is an increasing interest in developing derivative products for the public. A recent review of existing programmes for patient and public involvement in guidelines found that almost half of the reports indicated that patients were involved in the development of products specifically for patients and the public [1]. In addition, there are now many organisations producing patient versions of guidelines. In the UK, for example, the National Institute for Health and Care Excellence (NICE) and the Scottish Intercollegiate Guidelines Network (SIGN) produce freely accessible patient versions. The Finnish Medical Society, Duodecim, publishes patient versions of national Current Care guidelines at and a comprehensive collection of guideline-based patient information in Duodecim’s Health Library. Professional groups are also producing patient versions of their guidelines, for example, the Netherlands Association of Posttraumatic Dystrophy.

The research base for presentation and uptake of patient versions of guidelines is also growing. Much of the research draws on work about how to present evidence to patients in different formats – the GIN toolkit [2] for example - and how to develop decision aids from guidelines to promote the use and uptake of guidelines by patients and the public [3,4]. However, we know that other factors play an important role in the use of evidence and guidelines. Graham and Logan, for example, describe the characteristics of the patient as an important factor which could act as a barrier or facilitator to uptake [5]. These characteristics would include patient and public attitudes towards guidelines, and awareness of guidelines. The literature suggests, for example, that consumers may perceive guidelines negatively as a way to ration access to medications [6], a perception that would need to be addressed by material intended for the public.

The main objective of this review was to identify and synthesise evidence on the public’s attitudes towards clinical practice guidelines (including related patient versions) and evidence-based recommendations, as well as on their awareness. This work is part of a larger project which focuses on the communication of guidelines to a variety of target audiences in the DECIDE project (Developing and Evaluating Communication Strategies to support Informed Decision and practice based on Evidence: http://www.decide-collaboration.eu) [7].

## Methods

We conducted a synthesis of quantitative and qualitative studies similar to the approach used by Smith [8] and based on the methodology described by Dixon-Woods [9] and Munro [10]. We have reported this review using the guidance provided in the ENTREQ statement, an EQUATOR Network reporting guideline for the synthesis of qualitative research [11].

### Inclusion and exclusion criteria

We included quantitative and qualitative studies of any design reporting on the attitudes and awareness of guidance or guidelines, both for health care providers and patients and the public (patient versions). Other inclusion and exclusion criteria are given in Table 1.

**Table 1:** Inclusion and exclusion criteria

**Inclusion criteria**	**Exclusion criteria**
Public, patient or carer beliefs, feelings, awareness, understanding, knowledge, attitudes, expectations and perceptions of clinical practice guidelines (and/or guidance).	Opinion pieces, editorials, narrative reviews and protocols.
	Public/patient involvement in guideline development.
User-testing of public/patient information tools derived from guidelines.	Public/patient-centred communication/information not related to guidelines or evidence-based recommendations.
Readability/understandability of public/patient-targeted information materials derived from guidelines.	
	Public health campaigns.
Communicating research results to public/patients within the context of a guideline.	Procedure-specific information (e.g. details of surgical operations and their consequences).
Public/patient versions of guidelines	
Computer interpretable guidelines for public/patients.	Informed consent for clinical trials.
	Public understanding of science.
Knowledge translation tools for public/patients derived from guidelines.	
English, Finnish, Norwegian, Spanish and German articles.	

### Identification of studies

We searched MEDLINE, MEDLINE In Process, PSYCHINFO, ERIC, ASSIA from 2000 to January 2012 using key terms for patients, guidelines or guidance, awareness, perception, attitudes, communication and information dissemination (see Additional file 1 for the search strategies for principal databases). We updated our MEDLINE search up to January 2013. We conducted a search for secondary research in The Cochrane Library, Guidelines International Network (G-I-N) conference abstracts, Picker institute, Health Talk Online, Health Foundation, World Health Organisation, King’s Fund, Biomed Central, National Institutes of Health, NLM, AHQR, OpenDOAR, The Knowledge Network, NHS Evidence, TRIP database, Intute (up to January 2012), Google (including Google Scholar), Dogpile, and Health on the Net Foundation for documents published between 1999 and 2012. We also reviewed citations from key documents, authors and institutions (published between 1999 to 2012), and contacted experts in the field via emails to members of the DECIDE project, GRADE Working Group G-I-N and the Evidence Based Health discussion list (April 2012).

At least two authors independently screened each citation by title and abstract. We then retrieved the full text of all citations identified as potentially relevant by at least one investigator and two authors independently screened these full texts. Articles in English, Finnish, Norwegian, Spanish and German were included.

### Data extraction

Two authors independently extracted data from the included studies using a form which was first piloted and revised accordingly. Extracted data included study design and methods, recruitment strategy, study setting, number of participants, characteristics of participants (including age, sex, ethnicity, socio-economic status, and education level of participants), details of the interventions used to communicate guideline information, and awareness of clinical practice guidelines. From each study, we used an inductive approach to identify first order themes (i.e. themes based on the participants’ understanding reported by the authors) and second order themes (i.e. themes from the authors’ interpretation of the findings) related to attitudes and awareness of clinical practice guidelines.

### Quality assessment of studies

There is no agreed tool to assess the reliability of studies for qualitative research; we used the CASP (Critical Appraisal Skills Programme) tools [10]. Two reviewers independently assessed the methodological quality of the included quantitative and qualitative studies using the relevant CASP tool [12,13]. Studies were rated as good, fair or poor by considering all of the factors, but in particular, whether the study used a qualitative methodology appropriately to address our objectives.

### Data synthesis

Two reviewers compared the data extracted from all studies and resolved disagreements by discussion. We reported the awareness of guidelines for each study, and also a range across the studies, as we could not pool this data.

For the analysis of qualitative data, we based our analysis on approaches described by Smith [8], Dixon-Woods [9] and Munro [10] and conducted a thematic analysis [14]. Two reviewers first compared the themes extracted from each study to develop consensus. All themes (first and second order) were then compiled across the studies and the two reviewers organised the themes to develop categories of dominant themes with subthemes. Each paper was then recoded according to the categories of overarching and subthemes. This process was iterative with discussion between the two reviewers, and also involved consultation with the team. Quotes from the original studies were used to illustrate the themes.

## Results

### Selection of studies

**Figure 1:**
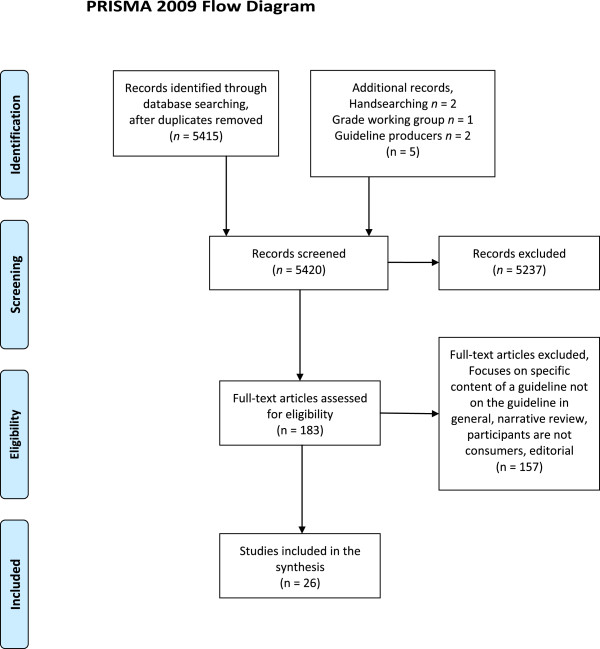
**PRISMA diagram of flow of studies.***From:* Moher D, Liberati A, Tetzlaff J, Altman DG, The PRISMA Group (2009). *P*referred *R*eporting *I*tems for *S*ystematic Reviews and *M*eta-*A*nalyses: The PRISMA Statement. PLoS Med 6(6): e1000097. doi:10.1371/journal.pmed1000097 For more information, visit http://www.prisma-statement.org.

We found 5415 unique records with the database search and five additional studies through other methods. We assessed 183 studies as potentially eligible and retrieved those in full text. After full-text screening, 26 studies, involving 24 887 individuals were included in the review (see Figure 1 for the PRISMA Diagram of flow of studies). Of the 26, 20 studies provided data for the thematic analysis and 17 studies provided data for the awareness of clinical practice guidelines.

### Study characteristics

There were ten qualitative studies using focus groups or semi-structured interviews [6,15-23]; thirteen cross sectional studies [24-36]; and three randomised controlled trials: [37-39].

Tables 2 and 3 provide the characteristics of the included studies. Overall the studies included diverse populations: Canadian office workers, female carers in Maryland, USA, Londoners attending drop-in centres in the UK for patients with mental health problems, visitors to a welfare centre in Seoul, women attending secondary care for menstrual abnormalities in Leicestershire, UK, and patients with Diabetes in Australia. The age of participants ranged from 30 to over 76 years, apart from one study on 11–15 year old adolescents [33]. Most studies included both genders although some included only women because of the topic (e.g. breast cancer).

**Table 2:** Characteristics of studies and themes identified in thematic analysis of attitudes to clinical practice guidelines (19 included studies)

**Author year**	**Aim**	**Participants and study location**	**Key themes identified by reviewers**
**Study design**			
**Quality**			
Akl 2007 [37]	To evaluate the use of symbols and words to present information on the strength of recommendations	84 participants, 64.1% female, 48.6% graduate - part of community health education programme; USA	Evidence behind recommendations; format issues
RCT			
Fair			
Berry 2010 [23]	To gain an understanding of public perceptions of Physical Activity guidelines put forward by a public health agency	22 participants in five focus groups, 18 to 70 years; Type II diabetes or cardiovascular disease; Canada	Patient as individual; format issues
Qualitative study			
Good			
Carman 2010 [6]	To determine how the concept of making health care decisions based on evidence of effectiveness could be translated into language that consumers would understand	34 consumers in 4 focus groups, 57 interviews and 1558 employees, 18–64; USA	Patient as individual; Guidelines control care; Guidelines as rules; Guidelines for physicians; Communicate with physician;Trustworthiness
Qualitative study			
Fair			
Crocetti 2004 [26]	To determine awareness and knowledge of infant feeding guidelines	102 Primary female caregivers mean age 27 years; 34% African American; 64% completed high school; Maryland, USA	Patient as individual
Cross sectional study			
Good			
Dykes 2004 [15]	To evaluate a tool to drive patient centred evidence based recommendations to facilitate guideline adherence	3 evaluators mean age 71 years, TV literate bedbound patients and carers (higher retirement income); Connecticut, USA	Self management; Format issues
Qualitative study			
Poor			
Eaton 2011 [38]	To determine whether an intervention based on patient activation and a physician support tool was more effective than usual care to improve adherence to National Cholesterol Education Program guidelines (USA)	4105 patients; primary care; mean age 52 control/54 intervention; 96% white; 59% female, southeastern New England, USA.	Communicate with physician, decision making; Self management; Patient as individual. Format issues
RCT cluster randomised			
Good			
Elad 2011 [27]	To gauge acceptance of 2007 American Heart Association guidelines on antibiotic prophylaxis after being notified about change by doctor	51 patients, 58 ± 17 yrs, 40% female with endocarditis; Israel.	Applicability of guideline information; Communicate with physician; Trustworthiness
Cross sectional study			
Good			
Faruqi 2000 [16]	To determine views, how to put into practice and disseminate clinical management of diabetes mellitus guidelines	5-20 participants recruited through Diabetes Australia in four focus groups; Sydney, Australia	Self-management; Communicate with physician; Guidelines for physicians; Format issues
Qualitative study			
Poor			
Geiger 2001 [17]	To determine awareness of dietary guidelines and test presentation formats	40 men and women (25–45); Missouri, USA	Guidelines for physicians; Guidelines control care; format issues
Qualitative study			
Poor			
Julian 2010 [18]	To determine knowledge and attitudes of women with menstrual disorders towards the use of evidence based clinical guidelines for their condition	24 women (22–54) attending secondary care; Leicestershire, England	Guidelines as rules; Guidelines control care; Guidelines for physicians; Patient as individual;
Qualitative study			
Good			
Keenan 2002 [29]	To examine knowledge and understanding and factors that influence knowledge - media/nonmedia/age and education	400 adults over 18 years old, 51.8% college degree, 56% female; Minnesota, USA	Format issues
Cross sectional study			
Fair			
McFarlane 2012 [30]	To determine public awareness of National Institute for Health and Care Excellence (NICE) guidelines and their implementation	1675 respondents (70% female, 61% (45–74 yrs old), 17% health care professionals); mostly England and Wales, UK	Guidelines for physicians; Guidelines control care
Cross sectional study			
Fair			
Michie 2005 [39]	To evaluate knowledge of guideline and take up when using behaviourally specific language	84 mental health users; 41–50 years; 51% women; London, UK	Format issues
RCT			
Poor			
Miroballi 2012 [31]	To determine awareness of infection control guidelines	1399 Cystic Fibrosis patients and their families, 38% patients, 62% family members; USA	Communicate with physician
Cross sectional study			
Fair			
Mitchell 2004 [19]	To determine knowledge of evidence based medicine and guidelines	33 patients with colorectal cancer and 9 carers, 43 to 86 years; 66% male, many had not completed high school; Austin, Victoria, Australia	Communicate with physician
Qualitative study			
Fair			
Owen-Smith 2010 [20]	To investigate patients’ and healthcare providers’ experiences of, and preferences for, implicit and explicit healthcare rationing	56 participants (31 patients, clinicians, healthcare managers); morbid obesity and breast cancer; Bristol, UK	Guidelines control care
Qualitative study			
Fair			
Quintana 2001 [21]	To explore how best to use the Internet to make evidence-based preventive health care guidelines available to physicians and consumers	39 participants (22 men, 17 women, 56% men), 35 to 65 years, experience using the Internet; Canada.	Format issues; self management; Trustworthiness; evidence behind recommendations; Communicate with physician; Decision making
Qualitative study			
Good			
Royak-Schaler 2008 [22]	To investigate patient-physician communication from the patient’s perspective about guidelines	39, age 30–75 (mean age 55), 72% college education, breast cancer survivors, all African American; Baltimore, USA	Self-management; Communicate with physician
Qualitative study			
Fair			
SIGN 2011 [34]	To investigate public awareness of Scottish Intercollegiate Guidelines Network (SIGN) guidelines and their implementation	239 respondents (66% female, 74% 45–74 yrs old, 61% had specific condition or disability); mostly Scotland, UK	Format issues; Evidence behind recommendations; Guidelines improve care; Guidelines for physicians
Cross sectional study			
Fair			
Squiers 2011 [35]	To assess how knowledgeable women were about the new recommendations in mammography	1221 women, 40–74, who had never had breast cancer; USA	Format issues; Evidence behind recommendations; Guidelines control care
Cross sectional			
Good			

RCT: randomised controlled trial.

**Table 3:** Characteristics of studies and results for studies reporting awareness of clinical practice guidelines (17 included studies)

**Author year**	**Aim**	**Participants and study location**	**Awareness**
**Study design**			
**Quality**			
Berry 2010 [23]	To gain an understanding of public perceptions of Physical Activity guidelines put forward by a public health agency	22 participants in five focus groups, 18 to 70 years; Type II diabetes or cardiovascular disease; Canada	Lack of awareness
Qualitative study			
Good			
Cameron 2007 [24]	To determine Awareness and Knowledge of Canadian Physical Activity Guide (CPAG) guidelines, prompted and unprompted	8892 adults aged 18 or older from Physical Activity Monitor; 52% female, 83% greater than high school education; Canada	4% aware of any guidelines for physical activity; 37% prompted aware of CPAG
Cross sectional study			
Fair			
Copeland 2005 [25]	To determine awareness of written guidelines that define which conditions require exclusion from the Child Care Centre	128 parents picking up children at Day Care Centre, 91% female, 69% African American; Baltimore City, USA	61% aware of guideline
Cross sectional study			
Fair			
Crocetti 2004 [26]	To determine awareness and knowledge of infant feeding guidelines	102 Primary female caregivers mean age 27 years; 34% African American; 64% completed high school; Maryland, USA	77% aware of guideline
Cross sectional study			
Good			
Faruqi 2000 [16]	To determine how to put into practice and disseminate clinical management of diabetes mellitus guidelines	5-20 participants recruited through Diabetes Australia in four focus groups; Sydney, Australia	Lack of awareness
Qualitative study			
Poor			
Geiger 2001 [17]	To determine awareness of dietary guidelines and test presentation formats	40 men and women (25–45); Missouri, USA	Lack of awareness
Qualitative study			
Fair			
Hong 2007 [28]	To determine awareness and knowledge of dietary guidelines	345 well people - 77% female; 46% <65 years. Randomly selected in one district Seoul urban population.	32.2% aware of dietary guidelines
Cross sectional study			
Poor			
Keenan 2002 [29]	To examine knowledge and understanding and factors that influence knowledge - media/non-media/age and education	400 adults over 18 years old, 51.8% college degree, 56% female; Minnesota, USA	45% aware of dietary guidelines
Cross sectional study			
Fair			
Mitchell 2004 [19]	To examine knowledge of evidence based medicine and guidelines	33 patients with colorectal cancer and 9 carers; 43 to 86 yrs old; 66% male; many had not completed high school; Austin, Victoria, Australia	No awareness
Qualitative study			
Fair			
Miroballi 2012 [31]	To determine awareness of Infection Control guidelines	1399 Cystic Fibrosis patients and their families; 38% patients, 62% family members in USA	65% aware of guidelines
Cross sectional study			
Fair			
Nash 2003 [32]	To determine cholesterol guideline awareness	1163 adults, 56% female, >40 years; Canada	32% (94/290) aware of guideline
Cross sectional study			
Poor			
McFarlane 2012 [30]	To determine public awareness of National Institute for Health and Care Excellence (NICE) guidelines and their implementation	1675 respondents (70% female, 61% (45–74 yrs old), 17% health care professionals); mostly England and Wales, UK	79% (824/1040) aware of guidelines
Cross sectional study			
Fair			
Owen-Smith 2010 [20]	To investigate patients’ and healthcare providers’ experiences of, and preferences for, implicit and explicit healthcare rationing	56 participants (31 patients, clinicians, healthcare managers); morbid obesity and breast cancer; Bristol, UK	Only 6/31 patients knew about NICE and what they did and 3 of these patients worked for health service.
Qualitative study			
Fair			
Roth 2010 [33]	To investigate knowledge of guidelines and if is this linked to following guidelines	1940 adolescents (11–15 yrs old); 49% female; England, UK	11% of children knew about the recommendations.
Cross sectional study			
Fair			
Royak-Schaler 2008 [22]	To investigate patient-physician communication from the patient’s perspective about guidelines	39 participants, 30–75 yrs old (mean age 55), 72% college education, breast cancer survivors, all African American; Baltimore, USA	Lack of awareness
Qualitative study			
Fair			
SIGN 2011 [34]	To investigate public awareness of Scottish Intercollegiate Guidelines Network (SIGN) guidelines and their implementation	239 respondents (66% female, 74% 45–74 yrs old, 61% had specific condition or disability); mostly Scotland, UK	64% (151/236) aware of guidelines
Cross sectional study			
Fair			
Spence 2002 [36]	To investigate awareness of Canada’s Physical Guide to Healthy Active Living	2719 participants; 18-76+ years; Alberta, Canada	20% (544/2719) aware of guideline
Cross sectional study			
Fair			

The qualitative research studies were mostly good to fair quality (Tables 2 and 3). Common reasons for fair quality were: lack of description or discussion about the analyses of the qualitative data reported in primarily quantitative studies; the role of the researcher and their relationship with the participants when conducting the focus groups or interviews; and whether or not saturation of data was reached. Most of the quantitative studies were also good to fair quality. Few included information about pre-testing questionnaires, or had poor response rates and/or high drop-out rates.

### Thematic analysis of the public’s attitudes towards clinical practice guidelines and evidence based recommendations

The thematic analysis of the included studies resulted in four overarching themes and sub-themes for the patient and public attitudes towards clinical practice guidelines:

•‘**Applicability of guidelines:** Patient as individual**,** Applicability of information to themselves

•‘**Purpose of guidelines for patient:** Communicate with physician**;** Decision making**;** Self-management

•‘**Purpose of guidelines for health care system and physician:** Guidelines control care (restrict/offer, access, cost)**;** Guidelines as rules

•‘**Properties of guidelines:** Format issues**;** Trustworthiness**;** Evidence behind recommendations

### Theme 1: Applicability of guidelines

Several studies reported that individuals expressed concern that guidelines may not personally help them and may not be applicable to their particular needs [6,18,23,26,27,38]. Two studies highlighted that treatment decisions should be tailor made to the individual and therefore guidelines may not be appropriate [6,18]. Although Julian [18] also indicated in a qualitative study of women with menstrual disorders that this may not be true for all patients and that:

Patients’ perception of clinical guidelines was also influenced by whether they viewed menstrual disorders as being unique to the individual patient and requiring personal treatment or as a process in which women experience similar symptoms requiring similar treatment.

Participants were inclined to trust their judgement based on their own unique experiences [6,26] or advice from others in similar situations rather than trust guidelines [18]. For instance, while the majority of women in a survey knew the guideline recommendations were *not* to give solid food to infants before four months, almost half did give their child cereal before the age of four months and more than 40% reported that the advice of a friend or family member was influential in this decision [26]. Participants also wanted to clearly see that guidelines could apply to them. When asked about a set of physical activity guidelines, participants indicated that they needed to identify with the guidelines first before reading or applying them [23] and this often lead to comments that personal stories should be included in guidelines to help people relate to the information [22,23]. However, guidelines were also seen as an affirmation of patient experiences. In one study, women with menstrual disorders saw the guidelines as a way of reducing the need to ‘prove’ that they really had a menstrual problem because their individual needs were being identified in the guidelines [18].

### Theme 2: Purpose of guidelines for patient

Nine studies described the potential purpose of guidelines for patients and the public and the role that guidelines have played to date [6,15,16,19,21,22,27,31,38]. Five studies reported that participants thought guidelines could be used as a simple tool to provide health information and recommendations which could lead to a better understanding of their health [15,16,21,22,38]. However, surveys conducted by SIGN and NICE found that only 8% of respondents thought guidelines were used to inform the public or dealt with advice for patients [30,34]. Patients also indicated that they could use guidelines to plan which questions they would ask their health care providers during the clinical encounter [6,16,19,21,22,27,31,38]. Guidelines, however, were not only useful for talking to doctors but they were also perceived as a tool that could be used independently, outside the consulting room. One study indicated that participants thought guidelines could help them make their own health care decisions [21] and in several studies, patients and the public identified guidelines as a source of information to manage their own care [15,16,21,22,38]. Breast cancer survivors felt guidelines could provide much needed recommendations regarding diet and physical activity [22]. Self- management was also important to diabetic patients who used guidelines to act as a good reminder for their own self-care [16]. Guidelines were not only considered useful for treatment but also for preventing disease [21]. Other studies reported that guidelines could also be used to ensure patients received the care to which they were entitled [20]; as a second opinion [21]; and as validation of their health problems [18].

There were however some concerns about the use of guidelines by patients. One study found that patients were worried that guidelines might impair the patient-doctor relationship by reducing confidence in the doctor and also through the potential to create conflict between patients and doctors [18]. Another study reported that patients felt that guidelines may take away decision-making from patients [6]. Finally there was concern about the trustworthiness of guidelines. In one study investigating the impact of recent modifications to an endocarditis guideline, if a patient’s doctor did not approve of the guideline changes then patients would not follow it [27].

### Theme 3: Purpose of guidelines for health care system and physician

Several studies indicated that patients thought guidelines had several purposes related to their care at a system level and the care provided by their health care providers [6,16,18,20,30,34,35,38]. Overall, participants’ feelings were mixed about whether guidelines affected their care positively or negatively.

In several studies, guidelines were seen as a way to keep health care providers up to date with current treatments [16,18] and also as a way to ensure consistent and high quality care [30,34]. The survey conducted by SIGN exploring the public’s understanding of the purpose of guidelines indicated that 44% thought guidelines to be ‘consistent best care/practice’ [34]. In a similar survey conducted by NICE, 11% of participants thought that the main purposes of guidelines was ‘Best care’, and 8% thought NICE ‘had something to do with fair access’ [30]. In addition, 20/24 (84%) of SIGN participants and 246/553 (45%) NICE participants felt more confident in their or their relative’s care and treatment as result of the relevant guideline being applied.

In contrast, many studies reported that participants thought guidelines may be rules that health professionals must follow rigidly [6,18]. Consequently, participants indicated that guidelines could lead to inflexibility in care provided to individual patients [18]; rationing or denial of care [6,18,35]; or limited access to innovative care that patients need [6]. Squiers found that participants felt that the breast screening guidelines may have been developed to restrict care or screening to particular groups, which can also lead to controversy if the guideline is misunderstood or controversial [35]. This viewpoint was also supported by the respondents to the NICE survey [30] with 11% believing cost effectiveness was one of the main purposes of guidelines.

### Theme 4: Properties of guidelines

A strong theme emerged in several studies: patients and the public emphasised the importance of formatting when trying to understand the guidelines and adapting the guidelines to themselves, and in how they perceived the guidelines. It was important to participants that the guidelines should be perceived as trustworthy. Berry *et al.* found that the simplistic format of the *Canada Physical Activity Guideline*, especially its use of cartoons, put people off [23] and that this undermined the guideline’s trustworthiness:

When it came to participants’ perceptions of [the guideline], they expressed a dislike for the cartoon-like format, which led some to actually question whether adults were the target audience and if the guide would be taken seriously.

Because of the use of cartoons participants felt they could not identify with the messages being put forward by the guideline.

Several studies found that a guideline’s usefulness was also related to whether it was engaging to read and could hold a person’s interest. Berry found that most participants thought the presentation of the guideline was dull and lacked the ‘glitz’ that would encourage people to pick up and read the guidelines [23]. Participants did not like to be presented with too much information [17] and liked information to be organised in layers (in particular on the internet), with quick access to layers of recommendations and the ability to drill down to get more detailed information [21].

Participants in many studies indicated that they wanted to know what to do and therefore the language needed to be clear and unambiguous [17,29,39]. People preferred simple phrases like ‘low in fat’ rather than more nuanced phrasing ‘balance your fat’ [17], and language that was specific and clear-cut [29]. Michie *et al.*[39] explored using ‘behaviourally specific plain English’ text, which had been amended using psychological theory to address potential barriers to implementing the guideline recommendations. This wording was perceived by patients and the public to lead “to stronger intentions to implement the guidelines, more positive attitudes towards them, and greater perceived behavioural control over using them” [39].

Two studies reported that variation in the quality of care, in research evidence and in treatment effectiveness, were genuinely new concepts for many people and it was unclear if guidelines were based on evidence [6]. Participants were unfamiliar with and sometimes confused by the terms ‘medical evidence’ , ‘quality guidelines’ and ‘quality standards’ [6]. Despite this confusion, several studies reported that participants expressed a strong preference to be informed about the quality of evidence (or certainty or uncertainty) that supports a recommendation [37]. In particular, participants preferred to know about uncertainty relating to outcomes of a treatment or test but were slightly more interested in knowing about uncertainty relating to benefits than harms [37].

### Narrative synthesis of patient and public awareness of guidelines

Seventeen studies focused on asking patients and the public about awareness of clinical guidelines, including those written for the public or professionals [16,17,19,20,22-26,28-34,36].

Awareness that clinical guidelines exist ranged from 79% to 0%. The largest numbers were found in the (824/1040) of respondents to a NICE survey [30] (which respondents entered through the NICE website) and 64% (151/236) (through the SIGN website) [34]. However, these results may represent awareness of an already ‘aware’ group of people. The smallest numbers were from few or no participants in focus groups when asked about their awareness of guidelines and/or guideline producers [16,17,19,20,23].

Other studies asked patients and the public about awareness of a particular guideline after implementing strategies to improve awareness [19,23,32,33,36]. Berry *et al.*[23] and Spence *et al.*[36], focused on *Canada’s Physical Activity Guide to Healthy Living*. Mitchell and White focused on the National Health and Medical Research Council colorectal cancer guidelines [19] and Nash *et al.* focused on national USA guidelines on managing cholesterol [32]. Whether or not participants were aware of a particular guideline, rather than guidelines in general, varied. Copeland *et al.* found in a survey that 61% (78/128) of parents were aware of illness exclusion guidelines from child care, though this was for any written guideline on illness exclusion rather than a named guideline [25]. Spence *et al.* found that 20% (544/2719) of respondents to their survey were aware of *Canada’s Physical Activity Guide to Healthy Living*[36] while Roth and Stamatakis found only around 11% of 1954 children aged 11–15 knew the key NICE recommendation for physical activity in children [33]. When asked about National Health and Medical Research Council colorectal cancer guidelines, none of 33 people with colorectal cancer taking part in interviews were aware of them, although all participants wanted a copy once they were made aware of them [19].

Keenan *et al.* examined consumers’ knowledge of the *1995 Dietary Guidelines for Americans* using a telephone survey of 400 adults in two cities and found that 55% of people had never heard of a document containing the government’s dietary guidelines [29]. Of the 180 who knew it existed, 119 could not name it and only one of those who gave a name identified the *Dietary Guidelines for Americans*. Hong [28]*,* found that 196/290 individuals interviewed about dietary guidance were not aware of the *Dietary Guidelines for Korean, a* sizeable minority (64/290) felt that dietary guidelines were unnecessary. Crocetti *et al.*[26] surveyed 102 female caregivers at their child’s 4 month well-child visit and found that 78% were aware of the guideline and the specific recommendation of when to begin feeding solids [26].

Miroballi surveyed 1399 people who had, or who were caring for someone with, cystic fibrosis about infection control guidelines from the Cystic Fibrosis Foundation in the USA [31]. Overall, 65% of respondents were aware of the guidelines but of these only 66% had discussed them with their care team.

Royak-Schaler ran four focus groups with 39 African American breast cancer survivors and found that participants wanted guidelines that could help them develop plans for follow-up and survivorship self-care [22]. This guidance was available, however participants were neither provided with it, nor aware of it.

## Discussion

The principal aim of the review was to identify and summarise patient and public attitudes to clinical practice guidelines. We found 26 studies of fair to good quality from which four main themes emerged: Applicability of guidelines, Purpose of guidelines for patient, Purpose of guidelines for health care system and physician, and Properties of guidelines. These themes represent patient and public attitudes to clinical practice guidelines which were written either specifically for health care providers or for patients and the public. We suggest that these themes may need to be incorporated into the design of patient versions of guidelines, to ensure their use.

For example, patients want to be seen as individuals with unique experiences and health care needs. The theme of Applicability to the individual, also known as ‘Personalisation’ or ‘Affiliation’ , refers to the problems people have identifying with information and understanding the relevance to them, or what does it have to do with ‘someone like me’ [40]. Additional research is showing that conveying information is more than a question of whether patients understand the statistical risks (e.g. 3 out of 100 people were cured), but also how patients can use the information in their own situations [41]. Presenting personal stories of real people with the same health care needs may be one way to connect the reader to the information in guidelines, although there remains the question of how to select stories: should there be an attempt to provide balance, or should stories focus on the positive (or negative)? Perhaps guideline developers should pursue partnerships with patient organisations and popular ‘patient story’ websites such as healthtalkonline or PatientsLikeMe to provide direct access from patient stories to relevant guidelines-derived material. Alternatively, providing ways for readers to tailor the information to themselves by using their own health information may help individuals apply guidelines to their own particular situation. Decision aids, which guide people through a decision while clarifying personal values, can be provided as supplementary resources linked to guidelines and can be semi-automated as demonstrated in the MAGIC guideline project for anti-antithrombotic therapy [42]. These guidelines, like others about Chronic Obstructive Pulmonary Disease, explicitly consider patient values and preferences when developing recommendations [43]; an approach the GRADE system has always considered when deciding the strength of a recommendation [44,45]. This is of particular importance in versions of guidelines intended to be used directly by the public.

Patients and the public also saw guidelines as potentially serving many purposes, such as being sources of health information, as tools for making decisions, or as a resource to manage their own care. Many guidelines, however, do not typically include background information about the conditions or the interventions covered in the guideline. This means it could be challenging for guideline producers to then develop patient versions as a source of general health information and it may require producers to dedicate additional resources to look outside the guideline for that information, even if only to signpost readers towards those other sources of information.

It is clear though that guidelines are different from other sources of health information: guidelines include evidence-informed recommendations about what should or should not be provided or done, something that other sources of information do not generally do. Thus the recommendations should not be lost when producing patient versions since these are what make guideline-derived material unique. But while some guidelines lend themselves to helping patients with recommendations about self-management (e.g. test your blood sugar daily), this may not be straightforward for other guidelines. Guideline producers committed to providing patient versions will need to consider each guideline individually to determine the intended purpose of the patient version.

Patients and the public did not always see guidelines in a positive light; we found that many consider guidelines as a way to ration and deny access to care. Guideline producers may need to overcome this barrier directly in the text of patient versions - perhaps by providing the evidence behind a recommendation to show where the recommendation came from, or to simply be explicit in saying that the aim of guidelines is not to ration care but to provide care based on the best evidence currently available.

This review also found that public awareness of clinical guidelines is low, linked to the perception that clinical practice guidelines are only for health professionals and have little or no relevance for patients or the public. An increase in the number of guideline producers developing patient and public versions of all or some of their guidelines may help to address this awareness issue. Guideline producers cannot assume that patients and the public will naturally go to their websites looking for high-quality information, or that they will ask health professionals about guidelines. The material will need to be easily picked-up by search engines, as well as being promoted to health professionals to hand to patients, before significant numbers of the public will be able to use it in their decision-making.

### Methodological limitations

The search was challenging as we could not filter our results by study design and our topic was broad (a problem also raised by others) [10]. However, we do not think we have missed any significant studies as we screened over 5000 citations and believe we have captured the most relevant studies for this review. We chose the widely used CASP tool to assess the quality of included studies, but we believe that it includes factors that may not be directly linked to the credibility of the results presented by the study (e.g. ethics approval). Therefore, studies scoring poorly on these factors may nevertheless have been higher quality which would further substantiate the results that we found. Regarding the degree of confidence we have in the synthesised results of this review, we have not provided an overall assessment. Although, there are methods to assess overall confidence in the quality of the evidence for reviews of interventions (e.g. GRADE), there is currently no agreed system to undertake this for syntheses of qualitative evidence. We have instead indicated that most results came from studies of good to fair quality and that the themes from this review of the literature may be important to consider when developing patient versions of clinical practice guidelines.

## Conclusions

Many guideline producers are producing patient versions of clinical practice guidelines. This review has found important factors, in addition to formatting issues, which may affect the uptake and use of these versions of guidelines by public, patients and carers. Guideline producers need to make clear how the information is relevant to the reader and how it can be used to make healthcare improvements. In addition, awareness of guidelines is generally low and guideline producers cannot assume that the public has a more positive perception of their material than of alternative sources of health information.

Research to develop and test a variety of methods to incorporate this information into patient versions of guidelines is currently being conducted in the DECIDE project (Developing and Evaluating Communication Strategies to support Informed Decision and practice based on Evidence: http://www.decide-collaboration.eu). This project aims to improve the way guideline information is presented to a wide range of stakeholders, including the public, patients and their carers [7]. The intention is that by addressing the public’s attitudes and awareness of clinical practice guidelines when producing versions of guidelines intended for the public, these will be more useful in supporting evidence-informed healthcare decision.

## Abbreviations

CASP: Critical Appraisal Skills Programme; DECIDE: Developing and Evaluating Communication Strategies to support Informed Decision and practice based on Evidence; ENTEREQ: Enhancing transparency in reporting the synthesis of qualitative research; G-I-N: Guidelines International Network; GRADE: Grading of Recommendations Assessment, Development and Evaluation; NICE: National Institute for Health and Care Excellence; PRISMA: Preferred Reporting Items for Systematic Reviews and Meta-Analyses; SIGN: Scottish Intercollegiate Guidelines Network.

## Competing interests

The authors declare that they have no competing interests.

## Authors’ contributions

All authors conceived of the review, screened studies, and read and approved the final manuscript. JH performed the searches. KL, MC and NS appraised the studies and abstracted data. KL and NS interpreted the data and wrote the review; ST also wrote the review.

## Pre-publication history

The pre-publication history for this paper can be accessed here:

http://www.biomedcentral.com/1472-6963/14/321/prepub

## Supplementary Material

Additional file 1 Search strategies for principal databases. Click here for file
