# Supplementary material for: Patient and public attitudes to and awareness of clinical practice guidelines: a systematic review with thematic and narrative syntheses
Source: BMC Health Serv Res. 2014 Jul 27;14:321. doi: 10.1186/1472-6963-14-321 (PMC4119247; doi:10.1186/1472-6963-14-321)
Supplement: Additional file 1 — Search strategies for principal databases. [file 1472-6963-14-321-S1.pdf]

Additional file 1

Search strategies for principal databases

OVID Medline (2000 to January 2012 - updated January 2013)
Medline in Process (2000 to January 2012)

1 exp Patients/
2 *Practice Guidelines as Topic/
3 *Guidelines as Topic/
4 (guideline* or guidance).tw.
5 or/2-4
6 exp Communication/
7 Comprehension/
8 Information Dissemination/mt [Methods]
9 Health Knowledge, Attitudes, Practice/
10 Attitude to Health/
11 Knowledge/
12 or/6-11
13 1 and 5 and 12
14 patient*.tw.
15 (public or "lay person" or "lay people" or citizen*).tw.
16 ("service user*" or consumer* or reader*).tw.
17 ("care-giver*" or "caregiver*" or carer*).tw.
18 (famil* or spouse* or relative* or partner* or parent*).tw.
19 or/14-18
20 (communicat* adj3 (guideline* or guidance)).tw.
21 (comprehen* adj3 (guideline* or guidance)).tw.
22 (present* adj3 (guideline* or guidance)).tw.
23 (disseminat* adj3 (guideline* or guidance)).tw.
24 ((understand* or understood) adj3 (guideline* or guidance)).tw.
25 ((belief* or believe) adj3 (guideline* or guidance)).tw.
26 (attitude* adj3 (guideline* or guidance)).tw.
27 (aware* adj3 (guideline* or guidance)).tw.
28 (knowledge adj3 (guideline* or guidance)).tw.
29 (expect* adj3 (guideline* or guidance)).tw.
30 ((perception* or perceive) adj3 (guideline* or guidance)).tw.
31 ((inform or informing) adj3 (guideline* or guidance)).tw.
32 (accept* adj3 (guideline* or guidance)).tw.
33 or/20-32
34 *Practice Guidelines as Topic/
35 *Guidelines as Topic/
36 (guideline* or guidance).tw.
37 34 or 35 or 36
38 19 and 33 and 37
39 13 or 38
40 limit 39 to yr="2000 -Current"
41 Animals/
42 Humans/
43 41 not (41 and 42)
44 40 not 43

OVID ERIC (2000 to January 2012)

1. exp Patients/
2. exp *Guidelines/
3. (guideline* or guidance).tw.
4. 2 or 3
5. exp Communications/
6. exp "communication (thought transfer)"/
7. exp Comprehension/
8. exp information dissemination/
9. exp Knowledge Level/
10. exp Health Education/
11. exp Attitudes/
12. exp Beliefs/
13. exp Expectation/
14. exp Perception/
15. or/5-14
16. 1 and 4 and 15
17. patient*.tw.
18. (public or "lay person" or "lay people" or citizen*).tw.
19. ("service user*" or consumer* or reader*).tw.
20. ("care-giver*" or "care giver*" or caregiver* or carer*).tw.
21. (famil* or spouse* or relative* or partner* or parent*).tw.
22. or/17-21
23. (communicat* adj3 (guideline* or guidance)).tw.
24. (comprehen* adj3 (guideline* or guidance)).tw.
25. (present* adj3 (guideline* or guidance)).tw.
26. (disseminat* adj3 (guideline* or guidance)).tw.
27. ((understand* or understood) adj3 (guideline* or guidance)).tw.
28. (accept* adj3 (guideline* or guidance)).tw.
29. ((belief* or believe) adj3 (guideline* or guidance)).tw.
30. (attitude* adj3 (guideline* or guidance)).tw.
31. (aware* adj3 (guideline* or guidance)).tw.
32. (knowledge adj3 (guideline* or guidance)).tw.
33. (expect* adj3 (guideline* or guidance)).tw.
34. ((perception* or perceive*) adj3 (guideline* or guidance)).tw.
35. ((inform* or informing) adj3 (guideline* or guidance)).tw.
36. or/23-35
37. exp Guidelines/
38. (guideline* or guidance).tw.
39. 37 or 38
40. 22 and 36 and 39
41. limit 40 to yr="2000 -Current"

EBSCO PsychInfo (2000 to January 2012)

S58	S15 or S57 Limiters - Publication Year from: 2000-2012
S57	S4 and S21 and S56
S56	S22 or S23 or S24 or S25 or S26 or S27 or S28 or S29 or S30 or S31 or S32 or S33 or S34 or S35 or S36 or S37 or S38 or S39 or S40 or S41 or S42 or S43 or S44 or S45 or S46 or S47 or S48 or S49 or S50 or S51 or S52 or S53 or S54 or S55
S55	TI informing W3 guidance OR AB informing W3 guidance
S54	TI informing W3 guideline* OR AB informing W3 guideline*
S53	TI inform* W3 guidance OR AB inform* W3 guidance
S52	TI inform* W3 guideline* OR AB inform* W3 guideline*
S51	TI perceive* W3 guidance OR AB perceive* W3 guidance

S50	TI perceive* W3 guideline* OR AB perceive* W3 guideline*
S49	TI perception* W3 guidance OR AB perception* W3 guidance
S48	TI perception* W3 guideline* OR AB perception* W3 guideline*
S47	TI expect* W3 guidance OR AB expect* W3 guidance
S46	TI expect* W3 guideline* OR AB expect* W3 guideline*
S45	TI knowledge W3 guidance OR AB knowledge W3 guidance
S44	TI knowledge W3 guideline* OR AB knowledge W3 guideline*
S43	TI aware* W3 guidance OR AB aware* W3 guidance
S42	TI aware* W3 guideline* OR AB aware* W3 guideline*
S41	TI attitude* W3 guidance OR AB attitude* W3 guidance
S40	TI attitude* W3 guideline* OR AB attitude* W3 guideline*
S39	TI believe W3 guidance OR AB believe W3 guidance
S38	TI believe W3 guideline* OR AB believe W3 guideline*
S37	TI belief* W3 guidance OR AB belief* W3 guidance
S36	TI belief* W3 guideline* OR AB belief* W3 guideline*
S35	TI understood W3 guidance OR AB understood W3 guidance
S34	TI understand* W3 guidance OR AB understand* W3 guidance
S33	TI understood W3 guideline* OR AB understood W3 guideline*
S32	TI understand* W3 guideline* OR AB understand* W3 guideline*
S31	TI accept* W3 guidance OR AB accept* W3 guidance
S30	TI accept* W3 guideline* OR AB accept* W3 guideline*
S29	TI disseminat* W3 guidance OR AB disseminat* W3 guidance
S28	TI disseminat* W3 guideline* OR AB disseminat* W3 guideline*
S27	TI present* W3 guidance OR AB present* W3 guidance
S26	TI present* W3 guideline* OR AB present* W3 guideline*
S25	TI comprehen* W3 guidance OR AB comprehen* W3 guidance
S24	TI comprehen* W3 guideline* OR AB comprehen* W3 guideline*
S23	TI communicat* W3 guidance OR AB communicat* W3 guidance
S22	TI communicat* W3 guideline* OR AB communicat* W3 guideline*
S21	S16 or S17 or S18 or S19 or S20
S20	TI (famil* or spouse* or relative* or partner* or parent*) OR AB (famil* or spouse* or relative* or partner* or parent*)
S19	TI ("care-giver*" or "care giver*" or caregiver* or carer*) OR AB ("care-giver*" or "care giver*" or caregiver* or carer*)
S18	TI ("service user" or consumer* or reader*) OR AB ("service user" or consumer* or reader*)
S17	TI (public or "lay person" or "lay people" or citizen*) OR AB (public or "lay person" or "lay people" or citizen*)
S16	TI patient* OR AB patient*
S15	S1 and S4 and S14
S14	S5 or S6 or S7 or S8 or S9 or S10 or S11 or S12 or S13
S13	MM "Expectations"
S12	MM "Attitudes"
S11	MM "Awareness"
S10	MM "Consumer Attitudes"
S9	MM "Knowledge Level"
S8	MM "Health Knowledge"
S7	MM "Information Dissemination"
S6	MM "Comprehension"
S5	MM "Communication"
S4	S2 or S3
S3	TI (guideline* or guidance) OR AB (guideline* or guidance)
S2	MM "Treatment Guidelines"
S1	MM "Patients"
